# Five-element music therapy based on emotion classification to improve sleep in patients with cancer-related depression: a multi-group randomized controlled trial

**DOI:** 10.3389/fpsyt.2025.1633673

**Published:** 2025-09-18

**Authors:** Wenjun Wang, Yudong Sheng, Hongmei Xie, Jingtao Zhao, Yang Zhao, Rui Zhou, Nan Zhao, Yu Wu

**Affiliations:** 1 Xiyuan Hospital, China Academy of Chinese Medical Sciences, Beijing, China; 2 Institute of Clinical Basic Medicine of Traditional Chinese Medicine, China Academy of Chinese Medical Sciences, Beijing, China

**Keywords:** sleep, cancer, depression, emotion, five-element music, randomized controlled trial

## Abstract

**Background:**

Sleep disorders are very prevalent in cancer-related depression (CRD) patients, which seriously impacts their quality of life. But the curative effect of drugs is not ideal. Existing five-element music therapy (FEMT) is effective in improving sleep and emotion in cancer patients, but lacks attention to matching the patient’s subjective emotion to the music. In this study, we propose the innovative “FEMT based on emotion classification”, aiming to validate its effect on improving sleep and emotion in CRD patients, and to compare its efficacy with that of traditional FEMT.

**Methods:**

120 CRD patients were randomly divided into the emotional classification music group (ECMG), the traditional music group (TMG), and the no music group (NMG). The outcome index was the difference between the Pittsburgh Sleep Quality Index (PSQI), Hamilton Depression Scale (HAMD), and Hamilton Anxiety Scale (HAMA) on the 14th and 28th days and baseline. the exploratory indexes were 5-HT, IL-1 β, IL-2, and TNF-α.

**Results:**

Both ECMG and TMG significantly improved the total score of PSQI, HAMD, and HAMA. ECMG significantly improved sleep disorder on the 14th day, and improved both sleep disorder and daytime dysfunction on the 28th day.TMG improved overall sleep quality and daytime dysfunction on the 28th day.

**Conclusion:**

Both therapies can effectively improve sleep disorders and negative emotions in patients with CRD. The former takes effect faster, while the latter has a more stable curative effect. The two therapies can be used as clinical adjuvant treatment, and personalized intervention programs can be selected according to patients’ needs.

**Clinical trial registration:**

https://www.chictr.org.cn/index.html, identifier ChiCTR2200062181.

## 1 Introduction

Tumors can alter the local microenvironment and cellular activity to evade the immune system and meet metabolic demands; consequently, the collateral damage from the host response leads to issues such as fatigue and cancer-related sleep disorders (CRSD) (1–3). CRSD is one of the main accompanying symptoms of cancer, and the incidence rate in cancer patients is 42%-69% (4, 5). It is a subjective experience in which cancer patients have insufficient sleep duration and quality to meet normal physiological needs, often manifesting as delayed sleep onset, sleep maintenance difficulty, reduced total sleep time, or early morning awakening (6–12). There is a strong correlation between depressive symptoms and sleep disorders in cancer patients. Sleep disorders are usually more serious in cancer patients with depressive symptoms, but this trouble is often overlooked (13, 14). CRSD can start before or during cancer treatment, and last for several years after treatment, which is recognized as the second largest cancer-associated symptom (15). It can lead to depression, anxiety, fatigue, invasive pain, impaired immune function, decreased quality of life, and even increased cancer mortality (16–19). CRSD are usually treated with drugs, including benzodiazepine receptor agonists, antidepressants, antihistamines, and melatonin receptor agonists. However, due to limited curative effect and adverse side effects such as sedation, tolerance, cognitive impairment, psychological addiction and physical dependence, their use is limited (20, 21). Considering the cancer treatment cycle and the recurrence of the disease, non-drug intervention is particularly important (22). Currently, non-drug intervention therapies for CRSD mainly include cognitive behavioral therapy, mindfulness-based stress reduction therapy, mind-body therapy, aromatherapy, etc. However, they all have certain defects. For example, cognitive behavioral therapy has complicated treatment steps, high costs, and low patient compliance (23); mindfulness-based stress reduction therapy focuses more on treating depression and anxiety, and lacks long-term research on improving insomnia; mind-body therapy lacks professional guidance and standardized guidelines (24); aromatherapy may affect the efficacy of drugs and interfere with treatment (25–27). There is still an urgent need to find safer and more effective treatments for CRSD.

Music therapy is the process of utilizing music as a therapeutic tool to address psychological issues. It achieves the goal of helping individuals meet physical, emotional, cognitive, and social needs through the application of music and instruments (28). With the satisfactory results of various studies and investigations, the application of music therapy in improving individual mental state has attracted more and more attention (29). Studies demonstrate that music therapy is an effective method for alleviating sleep disturbances, improving negative emotions, and enhancing quality of life in cancer patients (30–36). Insomnia is a common symptom of various physical and mental illnesses, while music intervention provides a safe, non-pharmacological, and easily accepted treatment option (37, 38). Listening to specific soothing music can effectively shorten sleep onset latency, reduce nocturnal awakenings, prolong total sleep time, and improve sleep quality (39–42). Compared to pharmacological treatments, music therapy avoids potential drug dependence and side effects, while effectively alleviating anxiety and depressive emotions caused by insomnia. It creates a physical and mental state more conducive to sleep by regulating the autonomic nervous system and guiding attention (43, 44). In Western music therapy practice, the selection and application of music are typically based on modern psychological and physiological principles, as well as individual preferences, emphasizing the direct influence of musical elements such as rhythm, melody, harmony, and timbre on emotional and physiological responses (45). A defining feature of music therapy is the delivery of individually tailored music interventions conducted by trained and qualified music therapists. Therapists carefully select or improvise music tailored to patients’ needs, guiding activities such as listening, performing, creating, or discussing (46). It has been proven to have positive therapeutic effects on psychological or mental disorders such as depression, anxiety, insomnia, schizophrenia, autism, and dementia (47–52). The China-originated FEMT is a traditional Chinese music therapy, which is composed of five different scales: Jue, Zhi, Gong, Shang, Yu, which correspond to MI, SOL, DO, RE, and LA of Western music, respectively. In the “Five Elements” theory of traditional Chinese medicine (TCM), they symbolize five elements in nature: wood, fire, earth, gold, and water, which correspond to five organs: liver, heart, spleen, lung, and kidney. With each tone as the main tone, five different modes can be formed. TCM combines modes with body function, and gives listeners music with different modes according to the physiological laws and characteristics of the five internal organs and different symptoms of individuals. The interaction and resonance between the five tones and the five internal organs can improve the functions of the five internal organs, adjust people’s body and psychology, and thus affect the individual’s emotional state. Numerous studies have confirmed that FEMT, based on Traditional Chinese Medicine theory, can effectively improve sleep and mood, even surpassing conventional music therapy in some aspects. It is increasingly being introduced into the treatment of CRSD (53–55). However, in the past, FEMT of TCM mainly adopted a single method of listening to music by acceptance, which lacked attention to the relationship between subjective emotional feelings and musical emotions of the treated persons, which led to obstacles for some treated persons to enter the music scene and made it difficult to participate fully. The music emotion category is based on the subjective emotional feelings of the treated person to classify music, which is of great help to understand the emotional changes induced by music and help the treated person enter the musical imagination scene. Based on the traditional FEMT, our team innovated a FEMT for choosing music based on emotion classification. We included six experts in related fields (TCM, psychology, and musicology) to select music, analyze music emotions, classify them into emotional categories, establish music databases, carry out preliminary clinical verification, and finally complete this new FEMT. The purpose of this study is to determine whether this innovative FEMT has a positive impact on the sleep and emotion status of cancer-related depression (CRD) patients, and its clinical safety and feasibility. At the same time, its efficacy was compared with traditional FEMT in order to provide more accurate music intervention programs and evidence for sleep improvement and adjuvant treatment of cancer patients.

## 2 Methods

### 2.1 Design

This was a prospective, randomized, partially blinded, controlled, clinical trial. This experiment was approved by the Ethics Review Committee of Xiyuan Hospital, Chinese Academy of Traditional Chinese Medicine (Approval number: 2022XLA011-2), and has completed registration in the Chinese Clinical Trial Registry (registration number: ChiCTR2200062181). This study was conducted from March 2023 to September 2024. The subjects came from the oncology outpatient clinics and wards of two hospitals in Beijing (Xiyuan Hospital of China Academy of Chinese Medical Sciences and Guanganmen Hospital of China Academy of Chinese Medical Sciences). After fully explaining the research procedure, the subjects provided written informed consent. Evaluation and intervention were carried out in outpatient clinics and wards of the above two hospitals. FEMT is offered free of charge.

### 2.2 Participants

Participant screening and recruitment were conducted by a research assistant who was an experienced nurse.

The selection criteria were: (1) the disease was diagnosed as cancer; (2) Hamilton Depression Rating Scale (HAMD) score was≥ 8 and < 24; (3) age was ≥ 18 years old; (4) Karnofsky Performance Status (KPS) score was ≥ 60; (5) the expected survival time was more than 3 months;

Participants were excluded if they were: (1) patients with suicidal motives and suicidal tendencies; (2) patients with critical illness in the advanced stage of tumor; (3) patients with serious diseases such as heart, liver, kidney, and hematopoietic system disorders; (4) unconscious or mentally ill; (5) pregnant women; (6) people with hearing impairment or those who could not cooperate with treatment. During the treatment process, those who had poor compliance, failed to be treated according to the prescribed plan, and for whom the curative effect could not be evaluated were also eliminated.

The subjects were aware of the grouping situation, that is, they knew which group they belonged to.

### 2.3 Randomization and allocation concealment

An independent research assistant performed the randomization procedure. According to the randomized list (1: 1: 1), the eligible subjects were randomly assigned to three groups: Emotional Classification Music Group (ECMG), Traditional Music Group (TMG) and No Music Group (NMG). The randomization list was generated by an independent research assistant using SAS software before recruitment. Allocation criteria were defined such that all subjects had an equal probability of being assigned to each group, consistent with a simple randomization design. The group identifiers corresponding to the list were sealed in sequentially numbered opaque envelopes. The person who prepared and maintained custody of the envelopes was blinded, and she, along with the independent research assistant responsible for randomization, was not involved in any other steps of the study. After the participants completed the baseline assessment, the envelope was opened by the therapist.

### 2.4 Interventions

All participants were treated with routine regimens provided by oncologists. All therapists had more than 8 years of practical experience and had received professional training before the study began. We developed a “FEMT Software for Selecting Music Based on Emotion Classification”, which contained 144 music pieces screened and sorted by music experts (56). Based on the categories and tones of emotions, these music pieces were classified and constituted different music libraries.

ECMG received FEMT, which selected music based on emotion classification. In the first treatment, the therapist briefly introduced the treatment procedure. The treatment plan was based on the music theory of the five elements of TCM, evidence-based evidence, and clinical experience of treatment experts. The therapist guided the patients to use the software and enter the corresponding music library according to the disease diagnosis and TCM syndrome differentiation of the participants. Users could freely select 9 songs in the limited music library according to their preferences (each song lasted about 3 minutes). These songs belonged to the corresponding modes and emotional categories. After selection, the playing order was automatically arranged in strict accordance with the divided emotional categories to generate a personalized song list. The therapist informed the participants of the specific listening methods in detail, and asked the participants to wear headphones, lie down, close their eyes and listen to the selected tracks in sequence when they were relaxed, and to feel the music carefully. They listened for 30–40 minutes once a day for 4 weeks, and were treated 28 times in total.

TMG underwent traditional FEMT, following the same procedures as ECMG. However, the music library was limited to specific modes, with no emotional categories or specific order of playback. The participants were free to choose their own music. This listening session lasted 30–40 minutes daily for four weeks, for a total of 28 sessions.

**Figure 1:**
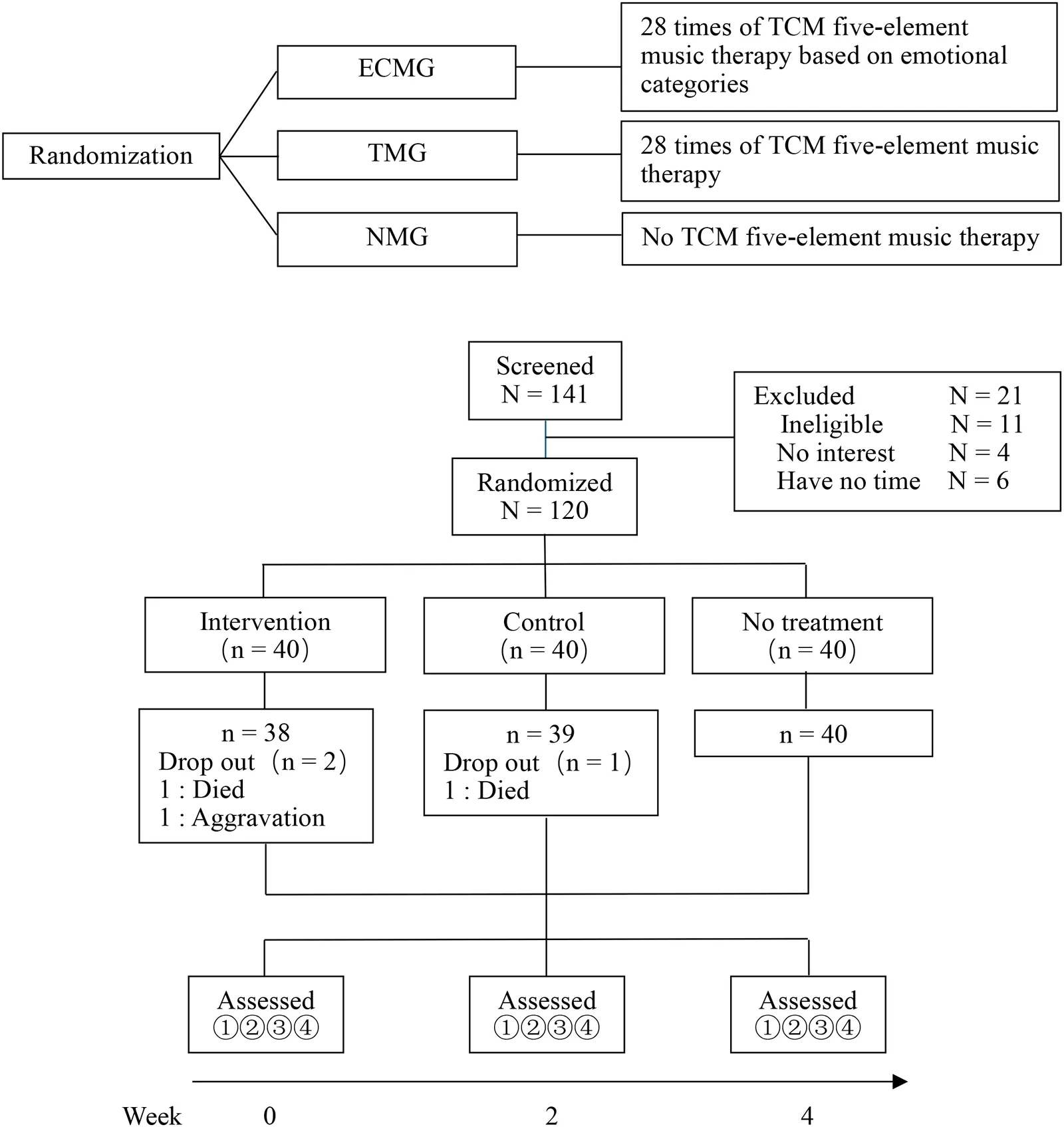
Research design and flow chart. 1 Hamilton depression scale (HAMD); 2 Hamilton Anxiety Scale (HAMA); 3 Pittsburgh Sleep Quality Index (PSQI); 4 Exploratory indicators (5-HT, IL-1β, IL-2, TNF-α).

NMG received routine treatment based on its condition, such as chemotherapy and radiotherapy (**Figure 1**).

### 2.5 Outcome measurement

The main outcome was the change of total score and 7 factor scores of the Pittsburgh Sleep Quality Index (PSQI) at baseline, 14th day, and 28th day. PSQI is an 18-item self-report questionnaire, which is divided into seven types of factors: subjective sleep quality, sleep latency, sleep duration, habitual sleep efficiency, sleep disturbances, use of sleeping medication, and daytime dysfunction. PSQI is used to evaluate sleep quality and disturbances, which has been fully verified in the diagnosis of CRSD (57). Secondary outcomes were the changes in the total scores of the HAMD and the Hamilton Anxiety Scale (HAMA) at baseline, day 14, and day 28. HAMD is a 17-item self-report questionnaire, which can be used to assess the severity of depression (58). HAMA is a 14-item self-report questionnaire, which is often used to diagnose and classify anxiety disorders (59). All the above scales were evaluated at baseline, day 14, and day 28. In addition, we also set up exploratory markers of 5-HT, IL-1β, IL-2, and TNF-α. Three groups of participants will collect 5mL of elbow venous blood on an empty stomach from 7:00 to 8:00 in the morning at baseline and after 4 weeks of treatment. After centrifuging with a table centrifuge at 3000r/min for 15 minutes, the supernatant will be taken, and the corresponding index content will be determined by professionals using an enzyme-linked immunosorbent assay. It is worth emphasizing that this measure was solely exploratory. Out of ethical considerations for cancer patients, sample collection was conducted only when subjects had a strong willingness to provide them and when physicians assessed their physical condition to be stable. Should there be any indication that the sample collection process might adversely affect the subject, it was prohibited.

Participants were asked if any adverse events (AE) occurred during each visit. The severity of AE is graded according to the AE General Terminology Standard V5.0. Any serious adverse events will be reported to the project researcher and ethics committee immediately. If too many serious adverse events occur, the trial will be stopped immediately.

The outcome assessor was blinded. She was unaware of the patients’ group allocation and conducted evaluations based on the patients’ random codes. Unblinding was performed only after all assessments had been completed. She was not involved in any other research procedures.

### 2.6 Data monitoring

This study was conducted by the Ethics Supervision Committee of Xiyuan Hospital, China Academy of Chinese Medical Sciences, for data and safety monitoring, including independent experts such as statisticians and clinical experts. They were not involved in the conduct of the study, but held regular meetings to review its progress.

### 2.7 Statistical analysis

Sample size estimation was performed based on the primary outcome measure (PSQI total score). A total of three groups were set up in this study. Using G*Power 3.1 software, an ANOVA model was selected for estimation. With the parameters set at f = 0.25, α = 0.05, power = 0.8, and considering an approximate 20% dropout rate, a total sample size of 120 cases (40 per group) was ultimately determined.

For participant characteristic data, an R×C contingency table and Cochran-Mantel-Haenszel (CMH) chi-square test were employed to analyze intergroup differences. Given that the difference in age distribution between groups was statistically significant, subsequent analyses of covariance included age as a covariate in all models to adjust for its potential influence. The analysis of covariance was based on the assumption of a Compound Symmetry (CS) covariance structure and was conducted entirely using SAS software (V 9.4). Because of the statistical significance of age distribution between groups, age was taken as a covariate for covariance analysis. One-way analysis of variance (ANOVA) was used to calculate the measured values of the total score of each scale and the PSQI at day 0, as well as the differences between day 0 and day 14, and between day 0 and day 28. Tukey’s test was used for pairwise comparisons. We also conducted power calculations with Welch’s test to reflect the credibility of the study results. Secondly, ANOVA was used to perform subgroup analyses for the changes in PSQI total scores by gender, cancer stage, and cancer type at day 0–14 and day 0-28. Mixed-effect models for repeated measures (MMRM) were used to conduct a type 3 test of fixed effects on each indicator score, to analyze the factors influencing each score, and to conduct within-group comparisons. Three subjects were excluded from the above analyses. They did not complete the evaluation program because they dropped out of the study. For biomarkers, this analysis was solely exploratory. Due to the considerable discrepancy in sample sizes before and after treatment and the lack of complete paired data, further robust analysis was limited. Therefore, listwise deletion was applied to missing data, and only the means and standard deviations of all available data were calculated. Due to the small sample size and the unpaired nature of the samples, only descriptive analysis was performed on the collected data, without inferential testing. This approach may introduce significant bias, and the results should be interpreted with caution.

In this study, three subjects withdrew after randomization but before receiving the first intervention assessment due to death or worsening condition These subjects did not receive any intervention and did not generate any follow-up data available for analysis. Therefore, we adopted the Per-Protocol (PP) analysis, aiming to evaluate the efficacy of the intervention protocol in the participants who actually completed the treatment most accurately.

All statistical tests were carried out using IBM SPSS Statistics 26, and a P value less than 0.05 was considered statistically significant.

A professional data processor conducted data analysis. The analyst was blinded and performed the analysis using group codes (e.g., experimental group = Group A). Unblinding was carried out only after all analyses had been completed. She was not involved in any other research procedures.

## 3 Results

### 3.1 Recruitment and baseline characteristics

From March 2023 to September 9, 2024, we screened 141 subjects for eligibility. 120 subjects were randomly divided into ECMG (n=40), TMG (n=40) and NMG (n=40). The baseline characteristics of the three groups are similar, and the cancer types cover a wide range (**Table 1**). Two participants dropped out of the study because of midway death, and one participant dropped out of the study because of aggravation of illness (the total wastage rate of the three groups was 2.5%). All intervention and control measures were implemented as expected. **Figure 1** is a research design and flow chart.

**Table 1:** Characteristics of participants.

Characteristic	ECMG (n=38)	TMG (n=39)	NMG (n=40)	F	P
Age, yrs	52.34 ± 11.61	55.69 ± 13.41	60.35 ± 12.79	3.96	0.0217
Educational attainment				0.02	0.8984
Undergraduate or above	13 (34.21)	6 (15.38)	8 (20)		
Post-secondary	9 (23.68)	3 (7.69)	3 (7.5)		
Secondary	14 (36.84)	28 (71.79)	17 (42.5)		
Primary or Below	2 (5.26)	2 (5.26)	12 (30)		
Cancer stage				0.48	0.4867
I	4 (10.53)	5 (12.82)	3 (7.5)		
II	6 (15.79)	6 (15.38)	9 (22.5)		
III	15 (39.47)	18 (46.15)	8 (20)		
IV	13 (34.21)	10 (25.64)	20 (50)		
Treatments				3.36	0.7619
Chemotherapy	17 (44.74)	20 (51.28)	20 (50)		
Radiotherapy	14 (36.84)	14 (35.9)	11 (27.5)		
TCM treatment	7 (18.42)	5 (12.82)	8 (20)		
Diagnosis				30.12	0.1807
Nasopharyngeal Carcinoma	0 (0)	0 (0)	1 (2.5)		
Colorectal Cancer	3 (7.89)	6 (15.38)	8 (20)		
Lung Cancer	2 (5.26)	3 (7.69)	3 (7.5)		
Buccal Cancer	0 (0)	1 (2.56)	0 (0)		
Oral Cancer	6 (15.79)	10 (25.64)	14 (35)		
Ovarian Cancer	2 (5.26)	0 (0)	4 (10)		
Breast Cancer	20 (52.63)	13 (33.33)	7 (17.5)		
Renal Cell Carcinoma	0 (0)	0 (0)	1 (2.5)		
Gastric Cancer	1 (2.63)	1 (2.56)	1 (2.5)		
Pleural Cancer	0 (0)	1 (2.56)	0 (0)		
Hematologic Malignancies	2 (5.26)	1 (2.56)	0 (0)		
Pancreatic Cancer	1 (2.63)	0 (0)	1 (2.5)		
Uterine Cancer	1 (2.63)	3 (7.69)	0 (0)		
PSQI Total Score	9.74 ± 4.46	8.67 ± 4.47	8.38 ± 4.18	1.04	0.3567
PSQI Fac1	1.68 ± 0.84	1.49 ± 0.68	1.43 ± 0.75	1.23	0.2967
PSQI Fac2	1.89 ± 1.01	1.74 ± 1.07	1.83 ± 0.93	0.22	0.8037
PSQI Fac3	1.13 ± 0.84	0.97 ± 0.9	0.93 ± 0.89	0.58	0.5602
PSQI Fac4	1.11 ± 1.16	0.95 ± 1.1	0.88 ± 0.91	0.48	0.6214
PSQI Fac5	1.55 ± 0.65	1.38 ± 0.54	1.3 ± 0.46	2.09	0.1288
PSQI Fac6	0.53 ± 1.08	0.62 ± 1.18	0.48 ± 0.91	0.18	0.8388
PSQI Fac7	1.84 ± 0.92	1.51 ± 0.94	1.55 ± 0.9	1.47	0.2334
HAMD Total Score	13.66 ± 4.46	12.77 ± 3.6	12.9 ± 4.18	0.53	0.5911
HAMA Total Score	8.5 ± 4.34	8.36 ± 4.78	8.23 ± 4.12	0.04	0.963

Data are presented as mean ± standard deviation or number (%).

PSQI, Pittsburgh Sleep Quality Index; fac1, subjective sleep quality; fac2, sleep latency; fac3, sleep duration; fac4, habitual sleep efficiency; fac5, sleep disturbances; fac6, use of sleeping medication; fac7, daytime dysfunction; HAMD, Hamilton Depression Scale; HAMA, Hamilton Anxiety Scale; ECMG, Classification Music Group; TMG, Traditional Music Group; NMG, No Music Group.

### 3.2 Main results

**Figure 2:**
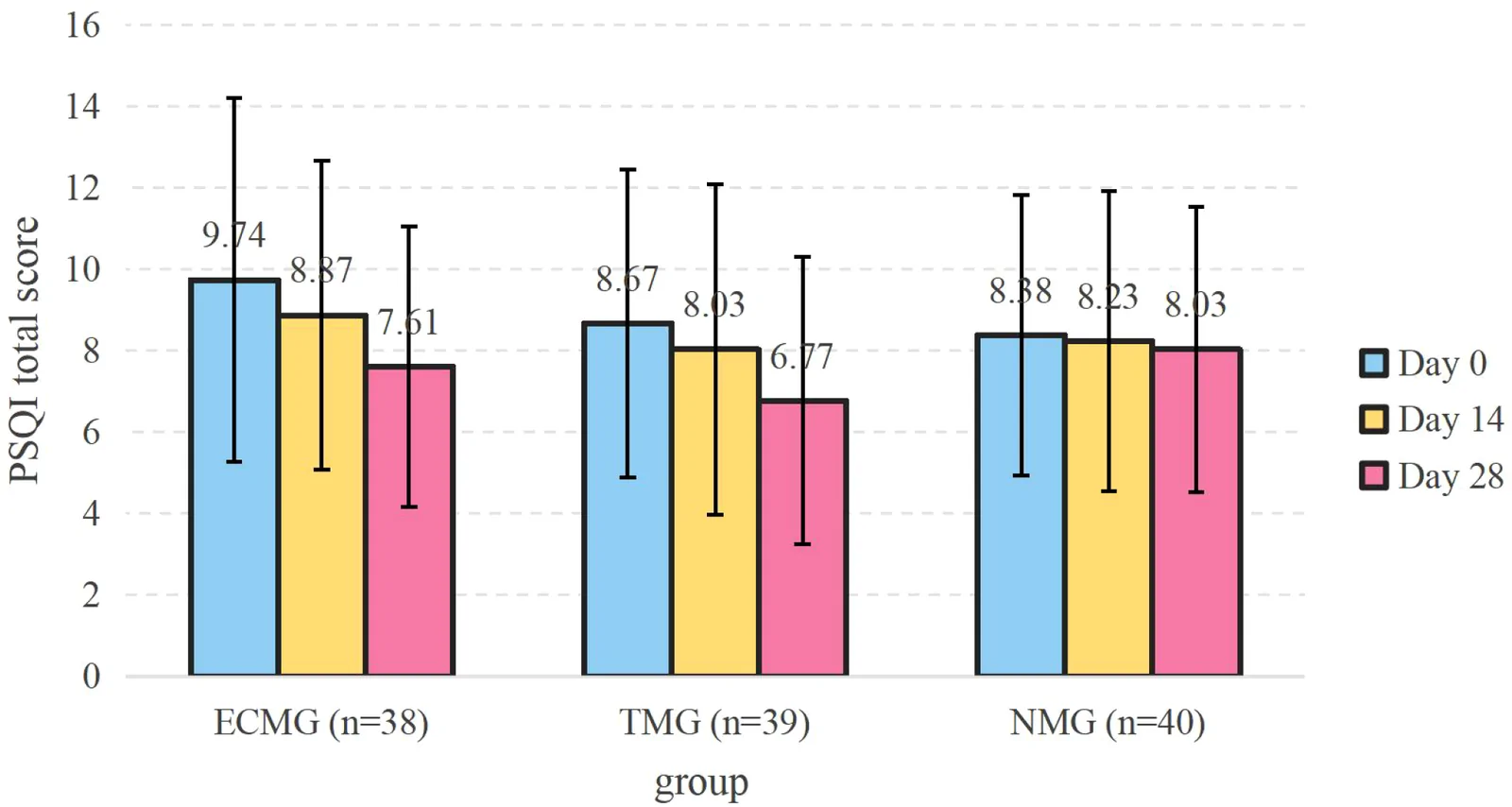
PSQI total score. Data are presented as mean ± standard deviation. Error bars represent SD (standard deviation). The sample size of each group remained stable at each time point (38/39/40 cases). PSQI, Pittsburgh Sleep Quality Index; ECMG, Classification Music Group; TMG, Traditional Music Group; NMG, No Music Group.

We made a statistical analysis of the total score and the 7 factor scores of PSQI. FEMT based on emotion classification and traditional FEMT have achieved better performance in improving PSQI total score, factor 5, and factor 7 scores. The difference in PSQI total scores from day 0 for TMG total scores at day 28 was greater than the difference from day 0 for NMG total scores (F = 3.58, P = 0.031, Pwr=0.68615) (**Figure 2**). Factor 5 on day 14, the difference between ECMG’s score and that of day 0 was greater than that of TMG and NMG (F = 6.84, P = 0.0016, Pwr=0.88104), and on day 28, ECMG and TMG were greater than NMG (F = 9.7, P = 0.0001, Pwr=0.97081). Factor 7 Day 0–28 difference ECMG and TMG are greater than NMG (F = 5.07, P = 0.0077, Pwr=0.91223). Factor 2 Day 0–14 difference ECMG is less than NMG (F = 3.16, P = 0.046, Pwr=0.62336). There is no difference in scores between groups at other time points (**Table 2**).

**Table 2:** Main results of the study: PSQI grade.

	ECMG (n=38)	TMG (n=39)	NMG (n=40)	ECMG vs TMG (MD[95%CI])	ECMG vs NMG (MD[95%CI])	TMG vs NMG (MD[95%CI])	F	P	Pwr	η²
Total score										
Days 0–14 difference	0.53 ± 2.98	0.64 ± 2.13	0.15 ± 2.01	/	/	/	0.45	0.6371	0.1483	0.01
Days 0–28 difference	1.79 ± 3.3	1.9 ± 2.46*	0.35 ± 2.81*	/	/	1.55 [0.01, 3.08]	3.58	0.031	0.6862	0.06
Fac1										
Days 0–14 difference	0.03 ± 0.72	0.08 ± 0.53	-0.05 ± 0.32	/	/	/	0.55	0.5802	0.2087	0.01
Days 0–28 difference	0.21 ± 0.7	0.36 ± 0.67	0.08 ± 0.47	/	/	/	2.06	0.1321	0.4678	0.03
Fac 2										
Days 0–14 difference	-0.08 ± 0.59*	0.28 ± 0.65*	0.05 ± 0.68	-0.36 [-0.71, -0.02]	/	/	3.16	0.046	0.6234	0.05
Days 0–28 difference	0.26 ± 0.83	0.33 ± 0.62	0.08 ± 0.8	/	/	/	1.24	0.2927	0.2610	0.02
Fac 3										
Days 0–14 difference	0.11 ± 0.83	0.05 ± 0.56	-0.03 ± 0.53	/	/	/	0.39	0.6752	0.1233	0.01
Days 0–28 difference	0.29 ± 0.8	0.21 ± 0.66	0 ± 0.82	/	/	/	1.5	0.2278	0.2885	0.02
Fac 4										
Days 0–14 difference	-0.03 ± 0.88	0.08 ± 0.77	0.03 ± 0.7	/	/	/	0.17	0.8478	0.0766	0
Days 0–28 difference	0.08 ± 0.97	0.33 ± 0.87	0 ± 1.06	/	/	/	1.26	0.2865	0.2839	0.02
Fac 5										
Days 0–14 difference	0.29 ± 0.52*^△^	0.05 ± 0.39*	-0.05 ± 0.32^△^	0.24 [0.01, 0.46]	0.34 [0.17, 0.56]	/	6.84	0.0016	0.8810	0.11
Days 0–28 difference	0.42 ± 0.55*	0.21 ± 0.41^△^	-0.05 ± 0.45*^△^	/	0.47 [0.22, 0.76]	0.26 [0.01, 0.51]	9.7	0.0001	0.9708	0.15
Fac 6										
Days 0–14 difference	-0.03 ± 0.59	0.05 ± 0.39	0.15 ± 0.7	/	/	/	0.92	0.4031	0.1772	0.01
Days 0–28 difference	0.08 ± 0.49	0.1 ± 0.88	0.3 ± 0.82	/	/	/	1.02	0.363	0.2345	0.01
Fac 7										
Days 0–14 difference	0.24 ± 0.63	0.05 ± 0.89	0.05 ± 0.45	/	0.50 [0.10, 0.89]	0.41 [0.01, 0.80]	0.96	0.3849	0.2719	0.02
Days 0–28 difference	0.45 ± 0.76*	0.36 ± 0.87^△^	-0.05 ± 0.55*^△^	/	/	/	5.07	0.0077	0.9122	0.08

Data are presented as mean ± standard deviation. */^△^ There was significant difference between the two data groups.

This power assumes the data will be analyzed with a significance level of 0.05.

fac1, subjective sleep quality; Fac2, sleep latency; Fac3, sleep duration; Fac4, habitual sleep efficiency, fac5, sleep disorder; Fac6, use sleep drugs; Fac7, daytime dysfunction; ECMG, Classification Music Group; TMG, Traditional Music Group; NMG, No Music Group.

Through the analysis of the mixed effect model, the comparison of each time point in the group, it was found that the total score, factor 5 score, and factor 7 score of PSQI on the 14th and 28th day after ECMG treatment were all lower than those on the 0th day. At the same time, TMG only decreased on the 28th day after treatment. There was no change in NMG. Using the type 3 test of fixed effects, it is found that the time factor influences factors 2, 3, and 6. Visiting time and the interaction between time and grouping influence total score, factor 5, and factor 7. Factor 4 is not affected.

Subgroup analyses for changes in PSQI total scores were conducted based on participant gender, cancer stage, and cancer type (**Table 3**). Gender was categorized into male and female. Cancer stage was classified into three categories: stages 1-2, stage 3, and stage 4. The cancer type was breast cancer. Changes between the three treatment groups were compared for the periods of Day 0–14 and Day 0-28. The results showed that, except for gender, the between-group differences in the other subgroups did not reach statistical significance. The gender subgroup analysis results indicated that the effect of FEMT Therapy on improving sleep scores differed by gender. Among male patients, there were no statistically significant differences in PSQI total score changes between the three treatment groups for either Day 0-14 (F = 0.49, P = 0.6156) or Day 0-28 (F = 1.55, P = 0.2311). Among female patients, the Day 0–14 changes showed no statistically significant difference between the three treatment groups (F = 0.41, P = 0.6662). However, the Day 0–28 changes reached a significant level (F = 3.32, P = 0.0408). TMG female patients showed the greatest magnitude of change (2.57 ± 2.25), followed by ECMG (1.68 ± 3.35), while NMG showed the smallest change (0.62 ± 2.47).

**Table 3:** Subgroup analysis: PSQI.

	ECMG(n=38)	TMG(n=39)	NMG(n=40)	TMG vs NMG (MD[95%CI])	F	P
Gender						
Male						
Days 0–14 difference	1 ± 0.82	0.09 ± 1.87	0 ± 1.92	/	0.49	0.6156
Days 0–28 difference	2.75 ± 2.99	0.18 ± 2.18	-0.14 ± 3.39	/	1.55	0.2311
Female						
Days 0–14 difference	0.47 ± 3.14	0.86 ± 2.22	0.23 ± 2.08	/	0.41	0.6662
Days 0–28 difference	1.68 ± 3.35	2.57 ± 2.25	0.62 ± 2.47	1.96 [0.15, 3.77]	3.32	0.0408
Cancer stage						
1 + 2						
Days 0–14 difference	0.09 ± 4.59	0.36 ± 2.69	0.75 ± 1.82	/	0.12	0.885
Days 0–28 difference	2 ± 4.1	1 ± 3.32	0.75 ± 2.49	/	0.44	0.6469
3						
Days 0–14 difference	1.36 ± 1.82	0.83 ± 1.98	0.75 ± 1.28	/	0.43	0.6568
Days 0–28 difference	1.86 ± 2.71	2.67 ± 1.91	1.5 ± 2.33	/	0.89	0.4204
4						
Days 0–14 difference	0 ± 2.2	0.6 ± 1.9	-0.45 ± 2.24	/	0.8	0.4551
Days 0–28 difference	1.54 ± 3.38	1.5 ± 2.01	-0.35 ± 3.07	/	2.13	0.1323
Cancer type						
Breast cancer						
Days 0–14 difference	0.38 ± 3.54	0.15 ± 2.41	0.43 ± 1.72	/	0.03	0.9713
Days 0–28 difference	1.71 ± 2.51	2.23 ± 2.71	1.71 ± 2.5	/	0.18	0.8372

Data are presented as mean ± standard deviation. */△ There was significant difference between the two data groups.

This power assumes the data will be analyzed with Welch’s test with a significance level of 0.05.

ECMG, Classification Music Group; TMG, Traditional Music Group; NMG, No Music Group.

### 3.3 Secondary results

**Figure 3:**
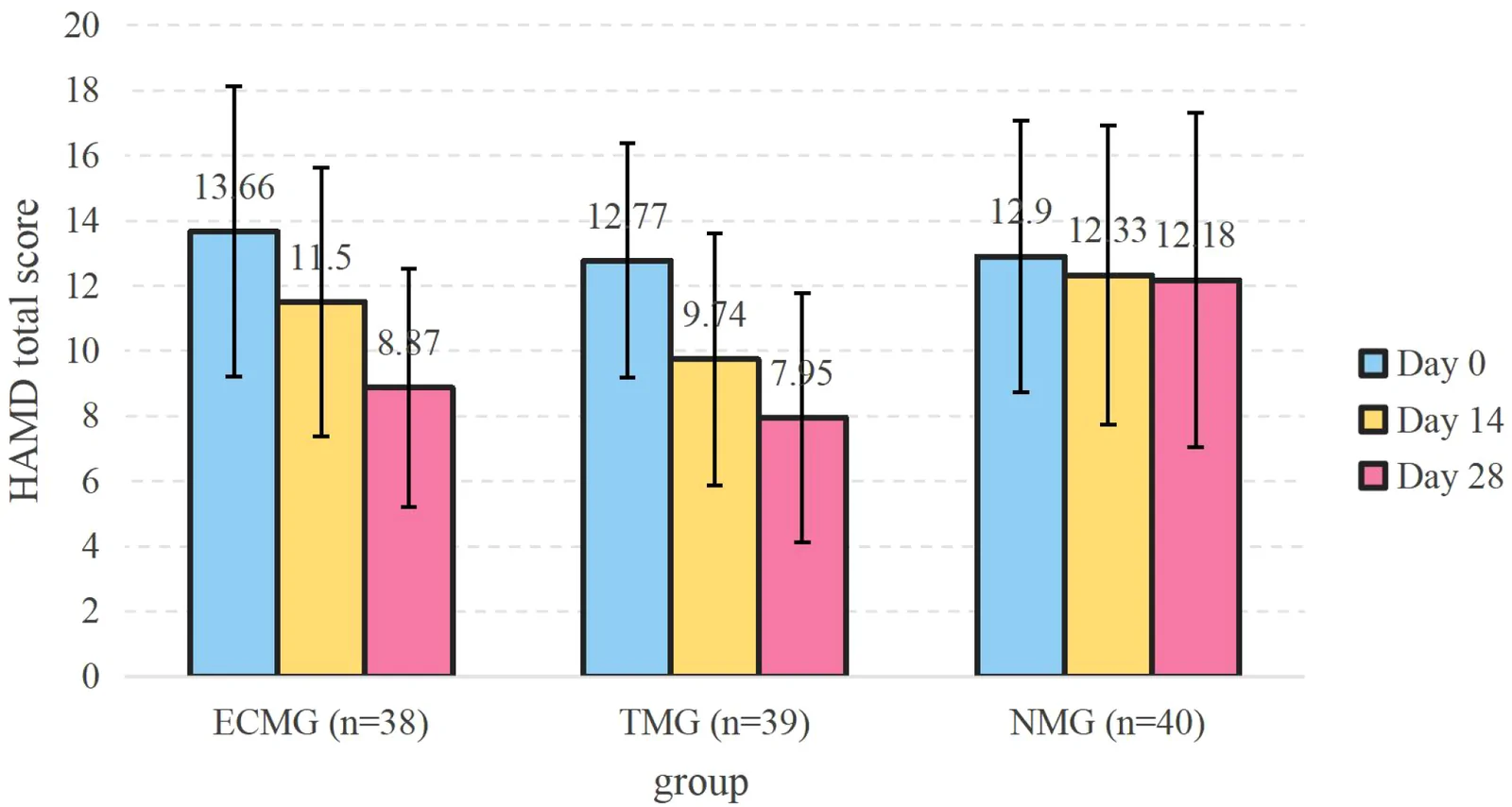
HAMD total score. Data are presented as mean ± standard deviation. Error bars represent SD (standard deviation). The sample size of each group remained stable at each time point (38/39/40 cases). HAMD, Hamilton Depression Scale; ECMG, Classification Music Group; TMG, Traditional Music Group; NMG, No Music Group.

**Figure 4:**
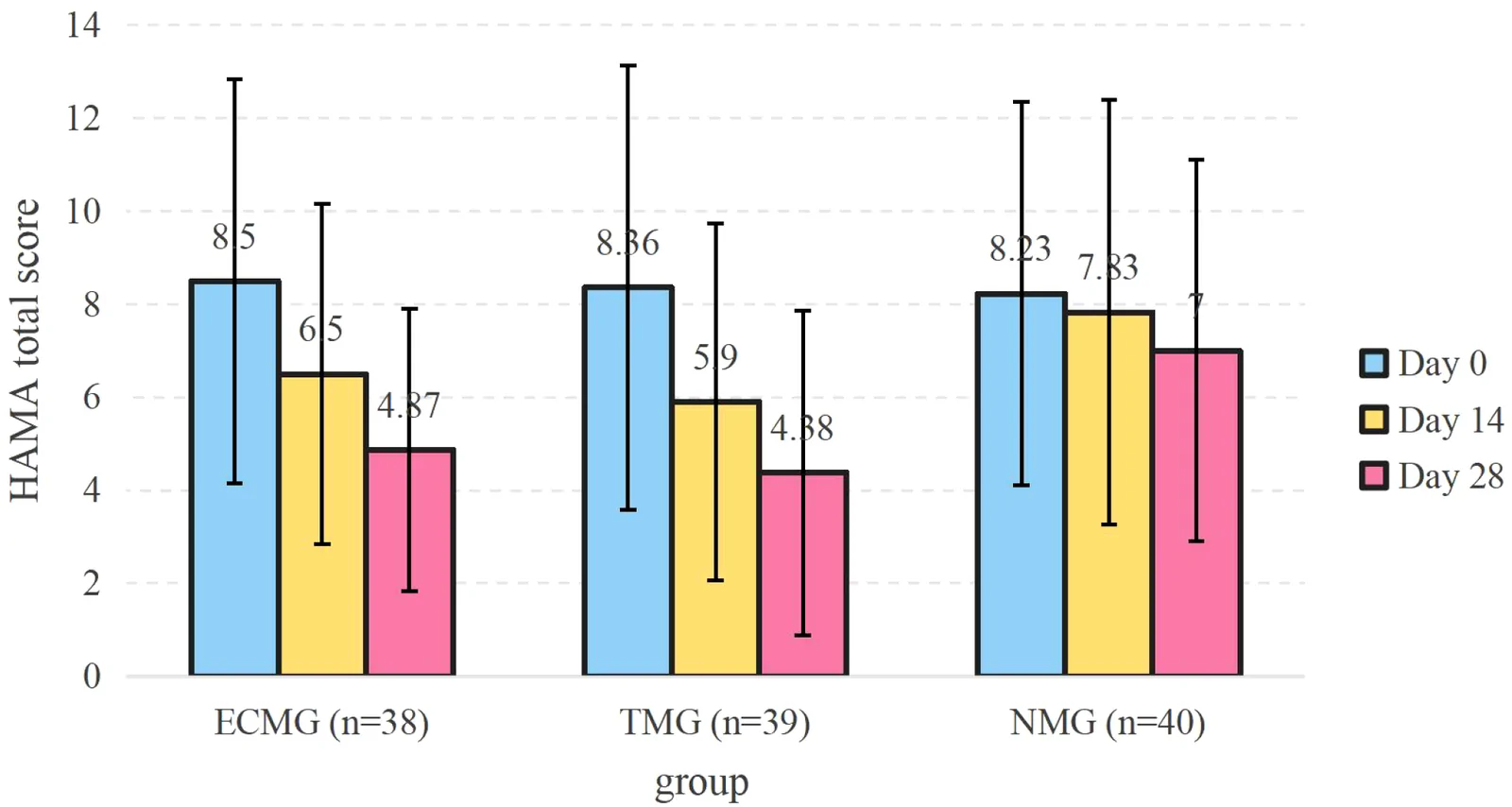
HAMA total score. Data are presented as mean ± standard deviation. Error bars represent SD (standard deviation). The sample size of each group remained stable at each time point (38/39/40 cases). HAMA, Hamilton Anxiety Scale; ECMG, Classification Music Group; TMG, Traditional Music Group; NMG, No Music Group.

Both FEMT based on emotional classification and traditional FEMT showed significant reductions in depression and anxiety scores (**Table 4**, **Figures 3**, **4**). On the 14th day, the total score of HAMD in ECMG and TMG decreased more than that in NMG (F = 9.81, P = 0.0001, Pwr=0.98091). There was no significant difference between ECMG and TMG. The same is true of the result on the 28th day. On the 14th day, the total score of HAMA in TMG decreased more than that in NMG (F = 4.95, P = 0.0087, Pwr=0.79248). There was no significant difference between ECMG and TMG. On the 28th day, the decreased values of ECMG and TMG were greater than those of NMG (F = 6.24, P = 0.0027, Pwr=0.93087).

**Table 4:** Secondary results of the study: HAMD total score, HAMA total score.

	ECMG (n=38)	TMG (n=39)	NMG (n=40)	ECMG vs NMG (MD[95%CI])	TMG vs NMG (MD[95%CI])	F	P	Pwr
HAMD total score								
Days 0–14 difference	2.16 ± 2.53*	3.03 ± 2.37^△^	0.58 ± 2.58*^△^	1.58 [0.24, 2.93]	2.45 [1.12, 3.78]	9.81	0.0001	0.9809
Days 0–28 difference	4.79 ± 3.5 *	4.82 ± 2.94^△^	0.73 ± 3.34*^△^	4.06 [2.31, 5.82]	4.10 [2.35, 5.84]	20.54	0.0001	0.1000
HAMA total score								
Days 0–14 difference	2 ± 2.6	2.46 ± 3.53*	0.4 ± 2.97*	/	2.06 [0.43, 3.70]	4.95	0.0087	0.7925
Days 0–28 difference	3.63 ± 3.3*	3.97 ± 4.67^△^	1.23 ± 3.17*^△^	2.41 [0.38, 4.44]	2.75 [0.73, 4.77]	6.24	0.0027	0.9309

Data are presented as mean ± standard deviation. */^△^ There was significant difference between the two groups.

This power assumes the data will be analyzed with a significance level of 0.05.

HAMD, Hamilton Depression Scale; HAMA, Hamilton Anxiety Scale; ECMG, Classification Music Group; TMG, Traditional Music Group; NMG, No Music Group.

Through the analysis of the mixed effect model, the comparison of each time point in the group, it was found that the total scores of HAMD and HAMA in ECMG and TMG on the 14th day and 28th day after treatment were lower than those on the 0th day. At the same time, the total scores of HAMD in NMG had no change, but the total scores of HAMA on the 28th day after treatment were lower than those on the 0th day. Using the Type 3 test of fixed effect, it was found that the visit time and the interaction factors between time and grouping influenced the total score of HAMD and HAMA.

### 3.4 Exploratory indicators

This study did not collect samples from all subjects. We obtained biological markers from 20, 18, and 13 subjects in the three groups, respectively, at intervention day 0, and from 13, 12, and 7 subjects in the three groups, at intervention day 28.

**Figure 5:**
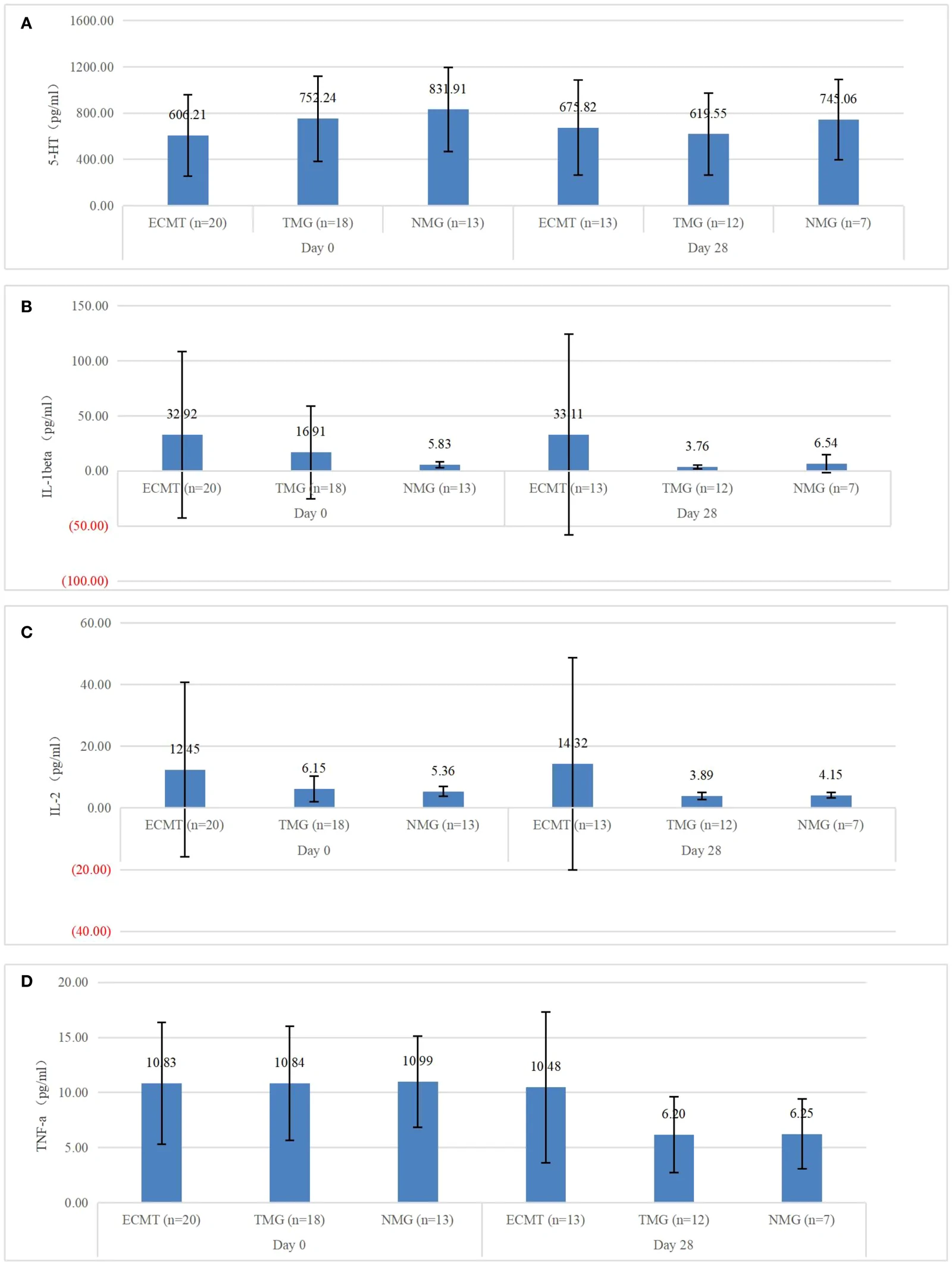
**(A)** 5-HT, **(B)** IL-1β, **(C)** IL-2, **(D)** TNF-α. Data are presented as mean ± standard deviation. Error bars represent SD (standard deviation). The sample sizes of each group were the same at different time points. The sample sizes at the baseline and on the 28th day were not equal. this analysis was solely exploratory. ECMG, Classification Music Group; TMG, Traditional Music Group; NMG, No Music Group.

After intervention, the levels of 5-HT and IL-2 in ECMG showed an upward trend, while the other two groups showed a downward trend. The level of IL-1 β in ECMG and NMG did not change significantly, but the level of IL-1 β in TMG decreased significantly. The levels of TNF-α in all three groups showed a downward trend, especially in TMG and NMG (**Table 5**, **Figure 5**). It must be emphasized that the above conclusions do not arise from rigorous inference; they only represent one possible trend, which can serve as a reference and exploration for subsequent related research. We strictly define the nature of these findings as hypothesis-generating. Our team will strive to continue exploring this area in subsequent research to obtain more rigorous data and results with higher credibility.

**Table 5:** Exploratory indicators: 5-HT, IL-1 β, IL-2, TNF-α.

	Day 0			Day 28		
	ECMG (n=20)	TMG (n=18)	NMG (n=13)	ECMG (n=13)	TMG (n=12)	NMG (n=7)
5-HT (pg/ml)	606.21 ± 351.90	752.24 ± 369.38	831.91 ± 362. 27	675.82 ± 410.79	619.55 ± 353. 06	745.06 ± 346.81
IL-1 β (pg/ml)	32.92 ± 75.77	16.91 ± 42.21	5.83 ± 2.75	33.11 ± 91.21	3.76 ± 1.80	6.54 ± 8.25
IL-2 (pg/ml)	12.45 ± 28.22	6.15 ± 4.10	5.36 ± 1.63	14.32 ± 34.43	3.89 ± 1.19	4.15 ± 0.92
TNF-α (pg/ml)	10.83 ± 5.52	10.84 ± 5.16	10.99 ± 4.14	10.48 ± 6.83	6.20 ± 3.45	6.25 ± 3.15

Data are presented as mean ± standard deviation.

ECMG, Classification Music Group; TMG, Traditional Music Group; NMG, No Music Group.

### 3.5 Adverse events

No adverse events occurred during the study.

## 4 Discussion

### 4.1 Main findings

This is a two-center, multi-group, randomized controlled trial. This study demonstrated that both emotion-based FEMT and traditional FEMT are effective in improving sleep disorders in depressed malignant tumor patients. The trial used a planned sample size based on the primary outcome measure, and baseline flattening was achieved through analysis of covariance The intervention variable was also singular, taking into account the expected dropout rate and the ability to detect differences in secondary outcome measures. Through *post-hoc* power analysis, we found that the primary outcomes (PSQI total score and factor scores) achieved high statistical power (Pwr > 0.68) in the actual data analysis, with some key indicators such as fac5 even exceeding 0.88 (**Table 2**). The secondary outcomes (HAMD and HAMA) also demonstrated very high statistical power (Pwr > 0.99), indicating that this study possessed sufficient statistical testing capability for both primary and secondary outcomes. Exploratory measures were excluded from these calculations due to their exploratory nature, though we conducted analyses to the best of our ability.

Emotion-based FEMT significantly improved sleep disturbances on day 14 of intervention and improved both sleep disturbances and daytime dysfunction on day 28. Traditional FEMT demonstrated significant improvements in overall sleep quality and daytime dysfunction on day 28. Both of them show different therapeutic advantages. FEMT based on emotion classification has a more obvious short-term curative effect and faster effect, while traditional FEMT has a more lasting curative effect. We speculate that this may be related to the playing order of emotion classification we set. In this experiment, we asked ECMG to listen to two kinds of music, namely, the sense of expression and the sense of talk, in order to induce participants’ emotions and guide them to enter the music scene faster. We ended with three kinds of music, namely, the sense of calm, freshness, and detachment, to calm down emotions or sublimate emotions. This can relieve the listener’s spirit and relax their body, so that they can easily enter deep sleep, reduce sleep interference, and accelerate the effect of FEMT based on emotional classification. In addition, according to the self-determination theory, enhancing individual choice autonomy may enhance the intrinsic motivation of treatment participation and then promote long-term treatment compliance (60, 61). Choosing the music playing sequence independently makes patients have better choices, so that the traditional FEMT shows a more stable cumulative effect of the overall curative effect in continuous intervention.

Unfortunately, FEMT based on emotion classification showed a very slight negative effect on sleep latency on the 14th day, although this turned into a positive effect on the 28th day. We guess that this may be because FEMT, based on emotional classification, adopts the step intervention of “emotional induction (expression/talk)-emotional calming (calm/detachment)”, which leads to short-term sympathetic dominance and increased physiological arousal level. However, the traditional FEMT may avoid continuous sympathetic activation because of its independent choice of playing sequence and random fluctuation of music stimulation intensity. With the continuous intervention of ECMG, parasympathetic regulation was gradually enhanced, and the balance of the autonomic nerves was reversed on the 28th day. But this guess needs to be verified by follow-up research.

Moreover, at day 28, females demonstrated better sensitivity to FEMT, especially towards the traditional FEMT. Previous relevant studies have mentioned that females exhibit higher enthusiasm and acceptance when using complementary therapies such as music therapy and show more significant effects in music therapy (62, 63). This may be related to females being more sensitive to hormonal levels and emotional fluctuations being more pronounced. It should be noted that these subgroup analyses are exploratory in nature and were underpowered. Potential confounding factors, such as uneven distribution of cancer types, may also be present. Therefore, these results should be interpreted with caution.

FEMT based on emotion classification and traditional FEMT have shown significant clinical efficacy in improving depression and anxiety of cancer patients, which proves that FEMT has unique therapeutic advantages in regulating the emotional state of cancer patients. It is worth noting that although the difference between the two groups is not statistically significant, the traditional FEMT shows a slightly better intervention effect than the FEMT based on emotion classification. We think this may be because although the structured playing of ECMG can accurately match the emotional categories, its fixed music sequence may interfere with patients’ immediate emotional expression needs and limit emotional mobility, thus having a certain impact on the curative effect.

In addition, exploratory indexes (5-HT, IL-1 β, etc.) did not show significant changes, which may be related to insufficient sample size or the selection of biomarkers. Due to patient compliance and other issues, we can’t collect biological samples from all personnel. These sample data also do not show a significant result orientation. Therefore, they can only be regarded as our exploratory research, and their significance still needs further verification. They can be used as a reference for future researchers to study the biological indicators of FEMT.

### 4.2 Research status

Existing studies have reported the significant impact of music therapy on cancer patients (64). Some studies have shown that FEMT, as one of the music therapies, has an optimistic clinical effect in improving emotional disorders, sleep quality, health status, and stress levels of cancer patients, and it is even more effective than Western music in regulating depression and anxiety levels (65–71). In addition, it also shows its feasibility, acceptability, and effectiveness for non-Chinese participants (71). Sleep disorders can be considered a very serious problem in cancer patients, especially those with depressive symptoms (72). However, music therapy studies for cancer patients mostly focus on emotional disorders of patients, and few studies pay attention to Depression with sleep disorders, a high-burden subgroup. Among the studies in this field, there are few high-quality clinical studies on the improvement of sleep disorders in cancer patients with depressive symptoms (73). In addition, the existing treatment mode of FEMT research is relatively single, which easily leads to the limitation of its clinical application scope and curative effect.

### 4.3 Research implications

Previous studies of Five Elements music therapy have primarily employed a relatively simple, receptive approach to music listening, lacking attention to the relationship between the patient’s subjective emotional experience and musical emotions. Categorizing traditional pentatonic music into emotional categories and adjusting the order of music playback based on the characteristics of each category not only leverages the unique characteristics of Five Elements music therapy but also highlights the correspondence between the patient’s subjective emotional changes and the musical emotion categories, making Five Elements music therapy more targeted and feasible. Our team’s proposed “FEMT for Music Selection Based on Emotional Classification” pioneered the integration of emotion classification into FEMT, constructing a personalized intervention framework of “emotion classification-music library optimization-music matching.” By systematically categorizing the emotional attributes of traditional Five Elements music and integrating its playback order, we explore more precise and effective methods for improving sleep and mood, which could provide innovative insights into the modernization of FEMT and the precision medicine paradigm. In terms of clinical validation, this study compared the efficacy of emotion classification-based FEMT and traditional FEMT through a multi-group randomized controlled design, clarified the added value of FEMT independent of conventional treatment, and verified its effectiveness and safety in improving sleep in patients with cancer and depression, providing an evidence-based basis for clinical promotion.

### 4.4 Limitations

This study has the following limitations and improvement directions: First, three subjects dropped out after baseline measurement and before the assessment began. As they had no evaluation data, we did not include them in the analysis. This may introduce selection bias, and the results may overestimate efficacy. In the future, more precise analysis should be conducted to avoid potential bias from such handling. Secondly, the intervention cycle designed by the study is relatively short, and it is difficult to evaluate the persistence of the curative effect due to the lack of long-term follow-up data. In the future, it is necessary to seek relevant clinical studies with a longer intervention cycle and make follow-up records to verify the long-term curative effect. Thirdly, due to ethical and clinical feasibility constraints, biomarker data were not fully collected, and *a priori* power calculations were not performed, limiting the depth of mechanistic exploration. In the future, we plan to use the results of this study as a reference for more rigorous and comprehensive biomarker studies, including stricter sample size design and longer-term data collection. Fourthly, although the evaluator blind method is adopted, it is difficult to eliminate the interference of the placebo effect by a partially blinded design. In the future, objective monitoring methods such as polysomnography should be combined to verify subjective report results and enhance the credibility of results. In addition, the research samples only included cancer patients from two 3A hospitals in Beijing, and the external validity was limited. In the future, we will try to carry out multi-center research and include cross-cultural samples for comparative analysis.

## 5 Conclusions

For patients with CRD, FEMT for 4 weeks has a good effect on sleep disorders and negative emotions. FEMT based on emotion classification and traditional FEMT can be used as an effective supplementary treatment to intervene in sleep disorders and negative emotions of CRD patients in the clinic. Their clinical efficacy has its emphasis, and appropriate therapy can be selected according to the actual situation.

## Data Availability

The datasets presented in this article are not readily available due to privacy and ethical issues. Requests to access the datasets should be directed to the corresponding author.
