# Correction: Five-element music therapy based on emotion classification to improve sleep in patients with cancer-related depression: a multi-group randomized controlled trial

**DOI:** 10.3389/fpsyt.2026.2004280

**Published:** 2026-10-15

**Authors:** Wenjun Wang, Yudong Sheng, Hongmei Xie, Jingtao Zhao, Yang Zhao, Rui Zhou, Nan Zhao, Yu Wu

**Affiliations:** 1 Xiyuan Hospital, China Academy of Chinese Medical Sciences, Beijing, China; 2 Institute of Clinical Basic Medicine of Traditional Chinese Medicine, China Academy of Chinese Medical Sciences, Beijing, China

**Keywords:** sleep, cancer, depression, emotion, five-element music, randomized controlled trial

Author “Yudong Sheng” was erroneously omitted as equal contributing author. Equal contribution marks have been added next to the names “Wenjun Wang and Yudong Sheng”.

A correction has been made to Section *2.4 Interventions*, paragraph 1. This initially stated the FEMT Software we used contained 153 music pieces; this should be “144”. The updated text reads:

“We developed a “FEMT Software for Selecting Music Based on Emotion Classification”, which contained 144 music pieces screened and sorted by music experts (56).”

A correction has been made to Section *3.4 Exploratory indicators*, paragraph 1. This initially stated that biological markers were collected from 20, 22, and 21 subjects in each of three groups at day 0 and from 13, 12, and 7 subjects at day 28; this should be “20, 18, and 13 and 13, 12 and 7” subjects, respectively. The updated text reads:

“This study did not collect samples from all subjects. We obtained biological markers from 20, 18, and 13 subjects in the three groups, respectively, at intervention day 0, and from 13, 12, and 7 subjects in the three groups, at intervention day 28.”

The original version of this article has been updated.

